# Supplementary material for: Disruption of the MreB Elongasome Is Overcome by Mutations in the Tricarboxylic Acid Cycle
Source: Front Microbiol. 2021 Apr 23;12:664281. doi: 10.3389/fmicb.2021.664281 (PMC8102728; doi:10.3389/fmicb.2021.664281)
Supplement: Supplementary file 1 [file Table_1.DOCX]

| **Strains** | **Relevant Genotype** | **Source** |
| --- | --- | --- |
| Keio Collection | *rrnB3* DE*lacZ4787 hsdR514* DE(*araBAD*)*567* DE(*rhaBAD*)*568 rph*-*1* | (Baba, Ara et al. 2006) |
| WT (BW25113) |  |  |
| *fumE* | *fumE::kan* |  |
| *icd* | *icd::kan* |  |
| *mdh* | *mdh::kan* |  |
| *gltA* | *gltA::kan* |  |
| *acnB* | *acnB::kan* |  |
| *sucA* | *sucA::kan* |  |
| *sucC* | *sucC::kan* |  |
| *sdhA* | *sdhA::kan* |  |
| *fumA* | *fumA::kan* |  |
|  |  |  |
| MG1655 | *rph1 ivgG rfb-50* |  |
| WT |  | Coli Stock Center |
| BB1 | *mdh::kan* | This study |
| *mrcA* | *mrcA::kan* | This study |
| *mrcB* | *mrcB::kan* | This study |
| RM602 | *mdh::frt; mrcA::kan* | This study |
| RM603 | *mrcB::frt; mdh::kan* | This study |
| RM605 | *mdh::kan*, pBad33-*mdh* | This study |
| RM606 | *mdh::kan*, pBad33 | This study |
| RM607 | pBad33 | This study |
| MreB_S14A_ | *mreB_S14A_::kan* | (Morgenstein, Bratton et al. 2015) |
| RM537 | *ftsZ-GFP_sw_::kan* | This study |
| RM609 | *ftsZ-GFP_sw_::kan; mdh::frt* | This study (modified from (Landgraf, Okumus et al. 2012) |
|  |  |  |
| Plasmids |  |  |
| pCP20 | Chl^r^, amp^r^, Flp recombinase, Rep101(ts) | (Cherepanov and Wackernagel 1995) |
| pBad33 | Chl^r^, pBAD | (Guzman, Belin et al. 1995) |
| pRM243 | pBad33-*mdh* | This study |
|  |  |  |

Table S1: List of strains and plasmids

| Strain | MIC_A22_ | MIC_Mec_ | MIC_Amp_ | MIC_Ceph_ | MIC_Cef_ |
| --- | --- | --- | --- | --- | --- |
| Keio Collection |  |  |  |  |  |
| WT | 1.5 ± 0.55 | 1.06 ± 0.39 | ND | ND | ND |
| *fumE* | 1.25 ± 0 | ND | ND | ND | ND |
| *icd* | 1 ± 0.31 | ND | ND | ND | ND |
| *mdh* | 20 ± 0 | ND | ND | ND | ND |
| WT | 1.25 ± 0 | ND | ND | ND | ND |
| *gltA* | 1.04 ± 0.36 | ND | ND | ND | ND |
| *acnB* | 10 ± 0 | 5.75 ± 0.87 | ND | ND | ND |
| *sucA* | 1.04 ± 0.36 | ND | ND | ND | ND |
| *sucC* | 20 ± 0 | 1.13 ± 0.39 | ND | ND | ND |
| *sdhA* | 1.04 ± 0.36 | ND | ND | ND | ND |
| *fumA* | 0.83 ± 0.36 | ND | ND | ND | ND |
| MG1655 |  |  |  |  |  |
| WT | 1.67 ± 0.65 | 0.41 ± 0.18 | 8.33 ± 3.23 | 35.83 ± 15.63 | 28.13 ± 10.27 |
| BB1 (*mdh*) | 20 ± 0 | 5.25 ± 1.84 | 11.46 ± 2.55 | 40 ± 15.49 | 10.16 ± 4.61 |
| RM605 | 7.81 ± 2.71 | ND | ND | ND | ND |
| RM606 | 35.94 ± 14.5 | ND | ND | ND | ND |
| RM607 | 5.63 ± 1.25 | ND | ND | ND | ND |
| *mrcA* | 20 ± 0 | ND | ND | ND | ND |
| RM602 | 20 ± 0 | ND | ND | ND | ND |
| *mrcB* | 2.5 ± 0 | ND | ND | ND | ND |
| RM603 | 2.81 ± 1.36 | ND | ND | ND | ND |

Table S2. List of MICs for strains tested. MIC_A22_- A22, MIC_Mec_- mecillinam, MIC_Amp_- ampicillin, MIC_Ceph_- cephalexin, MIC_Cef_- cefsulodin

ND: not determined

Figure S1. PCR of the *ftsZ* region. Primers bind ~50bp upstream and downstream of *ftsZ* (ftsZ.for – CGACGATGATTACGGC, ftsZ.rev – CTCGAAACCCAAATTCC). MG1655 and BB1 (*mdh*) have wild-type *ftsZ.* RM537 and RM609 contain *ftsZ-GFP_sw_*.

Figure S2. Deletion of *mdh* reduces cell area. A) Box plot of cell length measurements of individual cells grown to exponential phase. Data is pooled from three separate days of collection. Red pluses are outliers, the red line is the median, the bottom and top edges of the box represent 25^th^ and 75^th^ percentiles, respectively, and notches are 95% CI. Numbers shown are the mean cell length. p <0.0001 B) Average cell length of *mdh* complemented cells grown in 0.2% arabinose to exponential phase. Data is representative of an experiment done in triplicate. *** p <0.001 C-D) Cell width and area measurements of WT and *mdh* cells from A. E-F). Cell width and area measurements of WT and *mdh* complemented cells from B.

Figure S2. *sucC* does not cause a change in mecillinam resistance. MIC_mec_ of *acnB* and *sucC* mutants compared to WT type cells.

Baba, T., T. Ara, M. Hasegawa, Y. Takai, Y. Okumura, M. Baba, K. A. Datsenko, M. Tomita, B. L. Wanner and H. Mori (2006). "Construction of *Escherichia coli* K-12 in-frame, Single-gene Knockout Mutants: the Keio Collection." Molecular Systems Biology **2**: 2006.0008-2006.0008.

Cherepanov, P. P. and W. Wackernagel (1995). "Gene Disruption in *Escherichia col*i: TcR and KmR Cassettes with the Option of Flp-catalyzed Excision of the Antibiotic-resistance Determinant." Gene **158**(1): 9-14.

Guzman, L. M., D. Belin, M. J. Carson and J. Beckwith (1995). "Tight Regulation, Modulation, and High-level Expression by Vectors Containing the Arabinose PBAD promoter." J Bacteriol **177**(14): 4121-4130.

Landgraf, D., B. Okumus, P. Chien, T. A. Baker and J. Paulsson (2012). "Segregation of Molecules at Cell Division Reveals Native Protein Localization." Nat Methods **9**(5): 480-482.

Morgenstein, R. M., B. P. Bratton, N. Ouzounov, J. P. Nguyen, J. W. Shaevitz and Z. Gitai (2015). "RodZ links MreB to Cell Wall Synthesis to Mediate MreB Rotation and Robust Morphogenesis." Proceedings of the National Academy of Sciences: 12510-12515.
